# Lightweight Concretes with Improved Water and Water Vapor Transport for Remediation of Damp Induced Buildings

**DOI:** 10.3390/ma14195902

**Published:** 2021-10-08

**Authors:** Jaroslav Pokorný, Radek Ševčík, Jiří Šál, Lucie Zárybnická, Jaroslav Žák

**Affiliations:** 1 Department of Civil Engineering, Faculty of Technology, Institute of Technology and Business, Okružní 517/10, 370 01 České Budějovice, Czech Republic; jaroslav.pokorny@mail.vstecb.cz (J.P.); sal@mail.vstecb.cz (J.Š.); zak@mail.vstecb.cz (J.Ž.); 2 Institute of Theoretical and Applied Mechanics of the Czech Academy of Sciences, Prosecká 809/76, 190 00 Praha, Czech Republic; zarybnicka@itam.cas.cz

**Keywords:** lightweight concrete, alternative aggregate, compressive strength, remediation intervention

## Abstract

Most of the historical and old building stock in Europe are constructed from masonry, when brick, stones, or their combination are bound with traditional mortars. Rising damp, due to accompanying effects, is the main factor influencing the quality of indoor climate as well as having an important impact on the durability of masonry structures. In this study, new types of lightweight concrete with waste aggregate content as a suitable material for remediation of damp damaged masonries were designed and tested. Alternative aggregate served as silica sand substitution in the range of 0–100 vol.%. Basic structural properties, mechanical resistance, water, and water vapor transport properties were measured after 28 days of water curing and were compared with dense reference concrete and with traditional masonry materials as well. Moreover, the porous structure of produced concretes and changes caused by usage of alternative aggregate usage were evaluated with the mercury intrusion porosimetry (MIP) technique. Obtained experimental data showed the suitability of modified concretes with 25–50 vol.% of waste aggregate content to ensure acceptable strength and hydric properties, and these properties were found to be comparable with masonry structures and materials used in the past.

## 1. Introduction

Most of the building stock in Europe was being built during the 20th century as a consequence of recovery efforts after the Second World War [1]. Nowadays, many historical monuments and old masonry-based family and/or apartment buildings suffer from insufficient maintenance and rehabilitation and tend to undergo several decay processes that may lead to disruptions like sloughing elements, blistering, and flanking presence [1,2]. These aspects have clear causation with dampness coming from various sources, like wind-oriented rainfalls, condensation, capillary rise from the subsoil, etc. [3]

This study focuses on the rising damp that constitutes a still-discussed phenomenon causing the deterioration of indoor and outdoor appearances of historical as well as modern buildings built using traditional porous materials. This effect is closely connected with the presence and condition of horizontal water-proof insulations that can be absent or damaged [4]. Thus, damp may rise through a capillary system of porous materials [5,6] and due to its physical and chemical behavior under varying temperature and humidity conditions through the year. It may considerably support and accelerate degradation processes in structures connected with salts and chemical substances, freeze–thaw cycles, biological agents, wind-induced abrasion, and others leading to deterioration not only of aesthetical but also hygienic conditions of buildings [7,8,9]. At advanced deterioration stages, structures may be considerably disrupted, and serious damage to the object’s statics may occur [10].

So, the initial damages have to be repaired and rehabilitated as soon as possible to prevent other decay of structures caused by moisture [11]. In case of extensive damages of masonry structures, when some masonry elements/masonry parts are already absent or their strength properties are affected, extensive restoration works must be performed. In this view, some of the highly deteriorated structures are replaced with new suitable materials [12]. Jin et al. [13] presented the approach of crack remediation in brick masonry with the application of magnesium phosphate cements (MPC). MCPs are an alternative to the ordinary cement-based materials with significantly shortened setting times [14,15,16]. After the intervention, repaired fractured bricks had sufficient strength in a significantly shortened time in comparison to the usage of traditional cement-based repairing materials [13].

On the other hand, the long-term MPC resistance against water and moisture effect is questionable or not easily accessible [17,18]. Torres et al. [19] verified the effect of textile-based reinforced mortars intended for repairing and retrofitting masonry walls. Results showed high effectiveness in restoring the structural integrity of retrofitted structures, and the bearing capacity of treated structures under cyclic load was found to be two times higher. The substantial restoration efforts are dedicated to earthquake-impaired masonry structures [20,21]. In this connection, Khan and Ali [22] optimized application of reinforced-concrete stiffeners to confined brick masonry structures to effectively resist seismic loading. Horizontal and vertical stiffeners designed via diagonal approach were found to be the eligible solution in view of economical and seismic demands. In studies conducted by Ascione et al. [20] and Ghobadi et al. [21], fabric-reinforced mortars or stitched wire mesh were applied to infilled cement plasters which helped to restore the stiffness and ductility of damaged masonry. A specific approach has to be devoted in view of historical monuments. In this sense, San da Fonseca et al. [12] investigated bedding mortars in stone masonries, in order to design suitable repair mortar. In this context, the main attention has to be paid to ensuring adequate raw materials for preserving the heritage value of repaired structures. Furthermore, to choose such materials which will respect properties of originally used materials and, in addition, provide sufficient compatibility with fundamental substrates [12,23]. However, the proposed techniques and used materials do not always take into account additional aspects, such as increased dampness in structures. Repairing materials like MPCs and dense cement-based composites applied in structures or presented on their surface may create watertight barriers, which may cause additional moisture-based problems [11,24].

A considerable amount of old masonry-based buildings or historical monuments suffer from moisture problems, which can lead to further serious deterioration of the masonry’s state and durability. This paper is focused on the design and testing of lightweight concretes with alternative waste aggregate content and disposing of advanced liquid water and water vapor transport properties to be used for remediation of damp induced damaged buildings structures where masonry elements or even masonry parts are absent. Experimentally obtained data indicated a high rate of lightening of produced composites accompanied by growing porosity caused by the increasing amount of incorporated silica sand alternative. However, high porosity results in mechanical properties reduction. It was concluded that the optimized amount of LA ranged in the interval of 25–50 vol.%, when sufficient mechanical strength and improved hygric properties, comparable with traditional masonry materials (brick, sandstone, etc.), were ensured.

## 2. Experimental Part

### 2.1. Raw Materials

**Figure 1:**
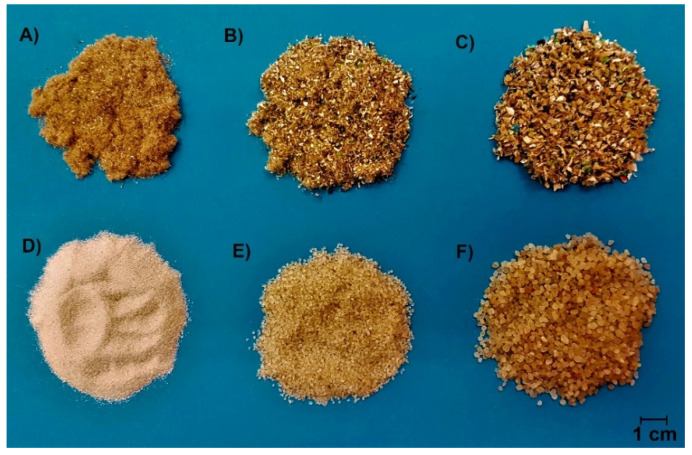
Samples of silica sand (SA) and waste aggregate (LA) separated into individual fractions; (**A**) LA (0.0/0.5 mm), (**B**) LA (0.5/1.0 mm), (**C**) LA (1.0/2.0 mm), (**D**) SA (0.0/0.5 mm), (**E**) SA (0.5/1.0 mm), (**F**) SA (1.0/2.0 mm).

**Figure 2:**
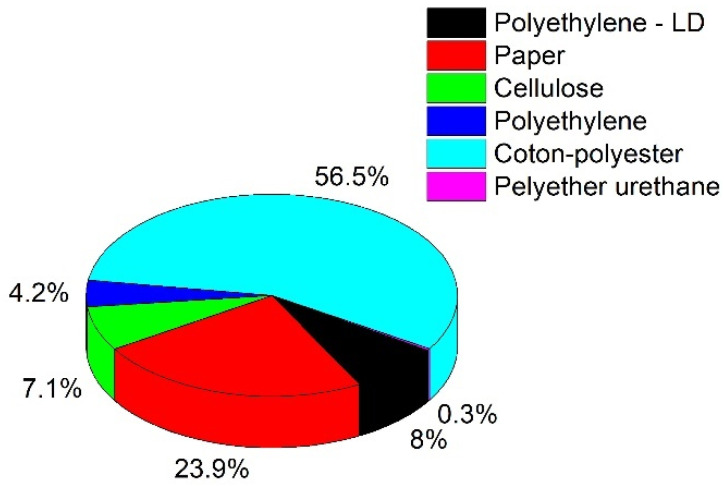
Composition of LA representative sample.

Ordinary Portland cement (OPC), meeting the strength class of 42.5 according to the standard EN 197-1 [25], manufactured by Českomoravský Cement, Ltd., Praha, Czech Republic, was used in this study. This cement type is specified for its chemical composition and material properties, i.e., powder density (EN 1097-3 [26]), specific density (EN 1097-7 [27]), and specific surface area (EN 196-6 [28]), and these properties are summarized in Table 1. A silica–sand blend (SA) used for concrete manufacturing was obtained from mixing of three separated fractions of 0.0/0.5 mm, 0.5/1.0 mm, and 1.0/2.0 mm with dosage in a weight ratio of 1:1:1. Lightweight aggregate (LA) represents fine ground waste from the interior reconstruction of buildings and substitutes natural aggregate. LA contained a balanced volume representation of the fractions of 0.0/0.5 mm, 0.5/1.0 mm, and 1.0/2.0 mm (see in Figure 1), and thus, its suitability as an SA replacing material could be considered. As mentioned above, LA constitutes are blended hardly to separate material whose components are listed in Figure 2. Due to the natural origin and characteristic material properties of cotton, paper and cellulose-based materials, a high absorption rate of LA blend for water can be assumed.

### 2.2. Mixing and Samples Casting

Five mixes, indicated in Table 2, with various alternative aggregate contents were prepared. The reference mixture contained the mass ratio of Portland cement (OPC) to natural sand (SA) and water corresponding to 1:3:0.5. In other cases, the natural aggregate was substituted with LA in volumetric ratio 0–100 vol.% with the step of 25 vol.%. For more clear interpretation, the batches of alternative aggregate were converted to mass ratio related to the cement proportion. The amount of added batch water had a decreasing tendency in order to ensure the constant value of spreading of 160 mm × 160 mm ± 5 mm. This need for water dosage reduction is related to very high liquid water absorption of waste aggregate when the average value of LA mix reached 368% after 24 h. Thus, LA has to be immersed in a water-filled vessel for 24 h before application as a mixing component. In the opposite way, dry LA addition, in particular regard to higher dosages, would cause the workability worsening, considering low water absorption of replaced silica sand is below 1%.

Input raw materials were homogenized in the laboratory mixing device E095 (Matest, S.p.A., Italy) according to the procedure specified in the standard EN 196-1 [29]. Fresh mixes were then cast into prismatic iron molds (40 mm × 40 mm × 160 mm), to conform with the standard EN 12390-1 [30]. Further, disk-shaped samples with a diameter of 110 mm and a thickness of 25 mm were made. After the first 24 h of hardening under polymer foil in laboratory conditions at a temperature of 20 ± 1 °C and 35 ± 5% of relative humidity, specimens were demolded and placed in a water environment for another 27 days.

### 2.3. Testing Procedures

Comprehensive material specification of both natural and waste aggregate was performed at the first stage of experimental investigation. The bulk density of aggregates in both loose and compacted states was determined using the method described in the standard EN 1097-3 [26]. The compaction of aggregate samples was performed on a vibrating table (Matest, S.p.A, Italy). Specific density was measured with the helium pycnometer AccuPyc II 1340 (Micro-Metrics Co., Ltd., UK), and the relative standard deviation of six replicates was calculated to be ≤0.05%. The procedure specified in the standard EN 1097-6 [31] was adopted for water absorption measurements performed on used aggregates. Particle size distribution of fillers was obtained performing sieving analysis described in the standard EN 933-1 [32]. The influence of waste aggregate on the properties of fresh mixtures was detected with the help of flow table measurement following the procedure specified in the standard EN 1015-3 [33]. On hardened samples (after a 28-day curing period), structural properties, mechanical performance, and water/water vapor transport ability were assessed. Bulk density measurements were carried out according to the standard EN 1015-10 [34], and the expanded uncertainty of the applied method corresponded to 2.8% for ten performed measurements. The open porosity was measured using the gravimetric method according to the standard EN 1936 [35] when prismatic samples 40 mm × 40 mm × 60 mm were placed inside the desiccator connected with a vacuum pump and left under vacuum treatment for 1 h at ambient temperature. After that, the desiccator was gradually filled with distilled water until tested samples were fully immersed. The filling rate was selected to 5 cm/h in order to ensure the better release of air bubbles [36]. The air pressure inside the desiccator was gradually increased to atmospheric value, and thereby intensive soaking of the liquid medium inside the porous structure of composites was allowed. The vacuum saturated mass of samples was repeatedly measured every 24 h until the constant mass of the samples was reached. The expanded uncertainty of open porosity determination was 5% (six independent measurements were carried out). Flexural and compressive strengths were measured on prisms with dimensions of 40 mm × 40 mm × 160 mm accordingly to the standard EN 1015-11 [37]. In this sense, the hydraulic press Servo Plus Evolution (Matest, S.p.A., Italy) with a loading capacity of 600/15 kN was used. Mentioned tests were always performed on a minimum of six samples. Thereby, the expanded uncertainty for both strength determination was ≤2%. The device DIO 562 (Starman’s Electronics, Inc, Czech Republic) was applied for non-destructive dynamic modulus measurements according to the method stated in the standard EN 12504-4 [38]. The expanded uncertainty of dynamic moduli determination was 1.6% attributed to six independent measurements. Porous space characterization of produced concretes was accessed with mercury intrusion porosimetry (MIP) method when Pascal 140 and Pascal 440 apparatuses were employed under the pressure of 140 kPa and 440 MPa, respectively. The rate of one-dimensional liquid water transport through the capillary system of basic silicates is specified with absorption coefficient (A) measured according to the procedure mention in the standard EN 1015-18 [39]. From known values of dry weight and capillary saturated weight of each sample, the capillary saturated moisture content (w_cap_) was calculated. Finally, the apparent moisture diffusivity (Χ_app_) was obtained as the relationship between the absorption coefficient and capillary saturated water content, as was reported by Luikov [40]. Tests were carried out on six specimens, and thus the expanded uncertainty of the applied method was about 6.5%. Water vapor transmission properties, such as water vapor permeability, a rate of passage of water vapor through the material, δ (kg·m^−1^·s^−1^·Pa^−1^), water vapor diffusion coefficient D (m^2^·s^−1^) and water vapor resistance factor µ (-) were determined with the use of the cup method formulated in the standard EN ISO 12572 [41]. Disk-shaped samples with a diameter of 105 mm and a thickness ranging from 20 to 25 mm were placed and sealed with silicon sealant in cups filled with activated silica gel to keep the relative air humidity of 3% or saturated water solution of KNO_3_ that provided a high relative humidity of 93% [42]. Both arrangement types were placed in the climate chamber ClimaCell Eco 222 (BMT Medical Technology, Ltd., Czech Republic) with a controlled temperature (23 ± 1 °C) and relative humidity (50 ± 3%) during measurements. The expanded combined uncertainty revealed for water vapor transmission tests corresponded to 2.5% (δ), 3.4% (D) and 2.3% (µ), taking into account six tested samples of every composite type.

## 3. Results and Discussion

### 3.1. Material Specification of Aggregates

The material characteristics of natural aggregate, as well as lightweight replacing alternative, are summarized in Table 3. To better understand the impact of silica sand replacement with waste blend, properties of three fractions, in intervals 0.0/0.5 mm, 0.5/1.0 mm, and 1.0/2.0 mm, respectively, were examined. The presented data clearly shows an almost 10-times lower bulk density in both loose and compacted states on behalf of waste material than traditionally used aggregate. Accordingly, these findings suggest a considerable reduction of weight of produced composites in the fresh as well as the hardened states. On the other hand, as is also evident from the mitigated density of LA due to its material composition (see Figure 2), a 460-times higher liquid water absorption rate after 24 h in water with respect to NA was detected, in particular, due to the increased content of voids. The ability of a lightweight aggregate to absorb water depends not only on its porous structure and interconnectivity of pores but also on the raw material/material blend from which it originates [43].

The particle size distribution of both aggregate types is presented in Table 4. Lightweight aggregate blend particle size distribution resembles that obtained for NA. However, the interval of 1.0–2.0 mm contains an alternative aggregate higher occurrence of rougher particles when the difference with regard to natural aggregate reaches 0.33 mm (d_50_) and 0.13 mm (d_90_). Only 10% of particles by weight are lightly finer in the case of LA. Differences in particle size distributions of particular materials are prescribed to the blended composition of waste aggregate with the predominance occurrence of plastic and paper-based components. The grinding of plastics can be a relatively complicated process in obtaining fine graininess because of their low melting point in dependence of plastic type [44].

### 3.2. Physical Properties

**Figure 3:**
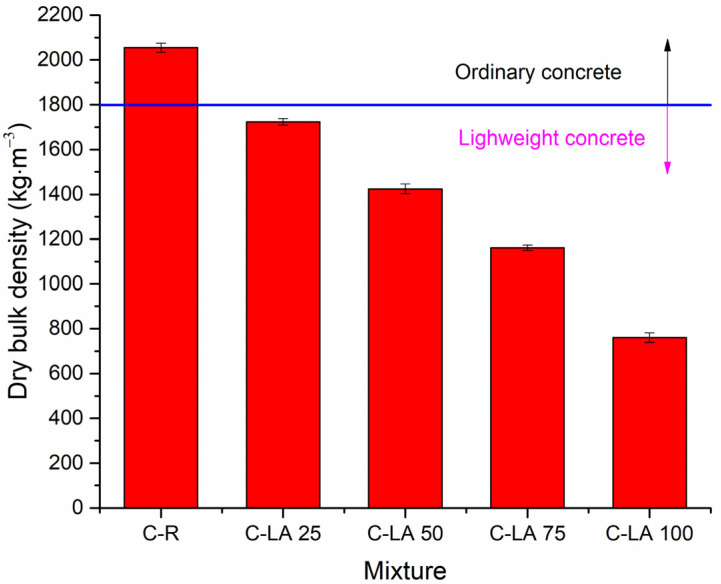
Dry bulk density of concretes in dependence on LA content. The value of 1800 kg·m^−3^ is highlighted and the distribution into two different concrete classes according to EN 206-1 [52] is inserted.

**Figure 4:**
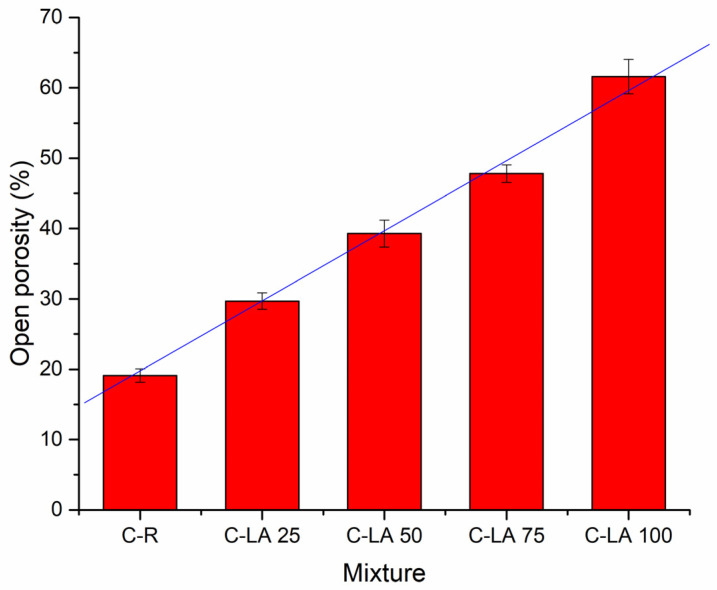
Open porosity development for reference and lightweight aggregate adjusted concretes.

Hardened concretes were, after 28 days of the curing period, subjected to structural properties determination. The dry bulk density development in dependence on lightweight aggregate content measured on produced composites is depictured in Figure 3. Increasing silica sand substitutions with LA led to a notable reduction of bulk density when values obtained from 2050 kg·m^−3^ (C-R), through 1720 kg·m^−3^ (C-LA 25), 1420 kg·m^−3^ (C-LA 50), and 1160 kg·m^−3^ (C-LA 75), up to 760 kg·m^−3^ (C-LA 100) were mitigated. From the indicated trend, one can note that all concretes with silica sand substitutions with LA can be included in the lightweight composites class. Similar observations were concluded in the study performed by Allahverdi et al. [45]. Expanded polystyrene (EPS) beads were applied to replace sand in lightweight concretes. The bulk density of produced composites was reduced from an initial 2100 kg·m^−3^ up to approx. 1250 kg·m^−3^ attributed to materials with 45% of EPS. The graphical illustration of open porosity development for different composites mixes is presented in Figure 4. Reduced bulk densities of LA dosed concretes induced a considerable gradual increase of open porosity with linear curve character (R^2^~0.99). In this context, open porosity of 19.1% was measured for the control material. For other samples with partial or full substitution of NA, porosity values were detected to increase with respect to C-R by: 10.6% (C-LA 25), 20.2% (C-LA 50), 28.7% (C-LA 75), and 42.5% (C-LA 100). It was reported that the porosity of historical bricks from the 19th century had open porosity of about 35% [46], and bricks produced during the 20th century has typically open porosity in the range from 32 to 38% [47,48]. In the case of sandstone, porosity may vary from very low at 16%, up to more than 40%, depending on the mining site [49,50]. Commonly used mortars for brick and stone masonries show the porosity of about 30% for those with the content of pozzolans or hydraulic binder and more than 40% for pure air binder [50,51]. In this view, presented values for masonry materials are in good conformity with those determined for composites C-LA 25 and C-LA 50 containing a waste alternative of silica sand in the portions 25 and 50 vol.%, respectively.

### 3.3. Mechanical Resistance

**Figure 5:**
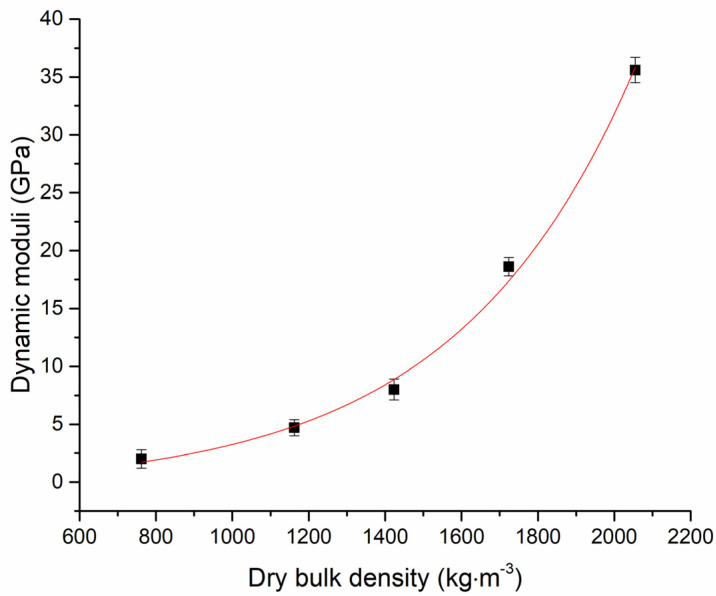
Dynamic moduli of fine-grained concretes with diverse LBW content.

Strength properties obtained for reference samples and concretes lightened with silica sand replacing aggregate are summarized in Table 5. As indicated by the increasing trend of open porosity, flexural as well as compressive strengths are gradually decreased as the silica sand proportion in concrete mixes was reduced. Measured flexural strength decreased from 8.0 MPa attributed to C-R, through 6.5 MPa (C-LA 25), 4.4 MPa (C-LA 50), 3.2 MPa (C-LA 75), up to 1.8 MPa corresponding to material with alternative aggregate content only (C-LA 100). That means a maximum of 4.4 times the flexural strength reduction. The substitution of natural sand up to 25 vol.% reduced the value of flexural strength only 1.2 times in comparison with C-R. The compressive strength value (determined as the average of six independent measurements for each type of composite) reached 56.6 ± 1.8 MPa for the reference sample. The incorporation of LA in the range of 25–100 vol.%, however, resulted in decrease of strength: 29.9 MPa (C-LA 25), 11.9 0.2 MPa (C-LA 50), 7.0 MPa (C-LA 75) and 3.3 MPa (C-LA 100), as summarized in Table 5. In the study conducted by Sengul et al. [53] expanded perlite, in this case, was applied to substitute a natural aggregate to produce the lightweight concrete. In that case, compressive strength decreased dramatically in dependence on growing perlite content—from 28.8 MPa of reference up to 0.1 MPa in the case of fully substituted traditional aggregate with perlite. In studies aimed to assess the compressive strength of old/historic brick and mixed masonries presented by, e.g., Sykora et al. [54], Matysek et al. [55] and Witzany et al. [56], mean compressive strength values around 20 MPa, but also in some cases decreasing up to 5.0 MPa [56] or 3.5 MPa [55] (with dependence on the used experimental technique and, in particular, the homogeneity of tested structures), were reported. In this context, waste aggregate filled concretes with a content reaching up to 25 vol.% could be considered as a good alternative to mixed masonries reaching average compressive strength values of about 20 MPa. Concretes with an LA aggregate representation higher than 25 vol.% and not exceeding 50 vol.% as a supplementary masonry material can be considered because of their lower compressive strength decreasing to 11.9 MPa. Dynamic moduli values with dependence on the dry bulk densities of produced concretes are plotted in Figure 5. The sharp decrease of dynamic moduli for the samples with lower dry bulk density is clearly visible. The biggest drop of dynamic moduli was determined to be in the range from 2000 kg·m^−3^ (C-R) up to almost 1400 kg·m^−3^ (C-LA 50). Overall, the huge reduction of dynamic moduli from approx. 35 GPa recorded for C-R to 2 GPa recorded for C-LA 100 was observed and such behavior clearly corresponds to the obtained compressive strength data mentioned above.

### 3.4. Porous Structure Specification

**Figure 6:**
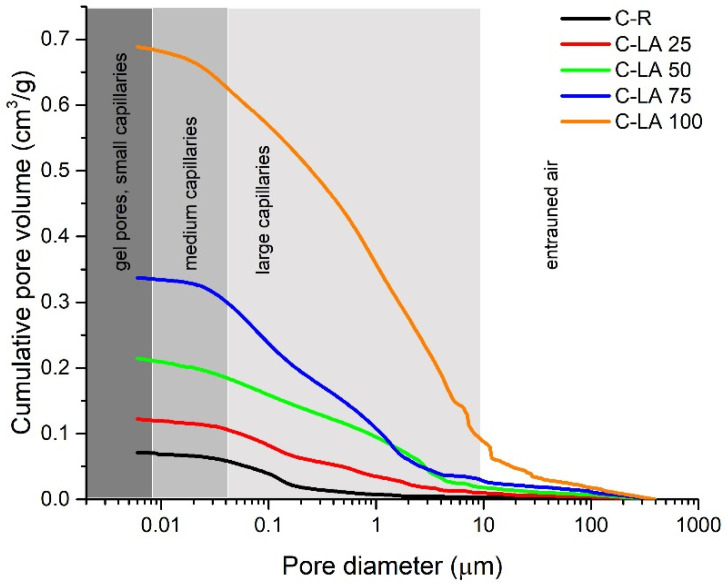
Pore size distribution-cumulative curves measured for control (C-R) and LA dosed samples (C-LA 25–100).

**Figure 7:**
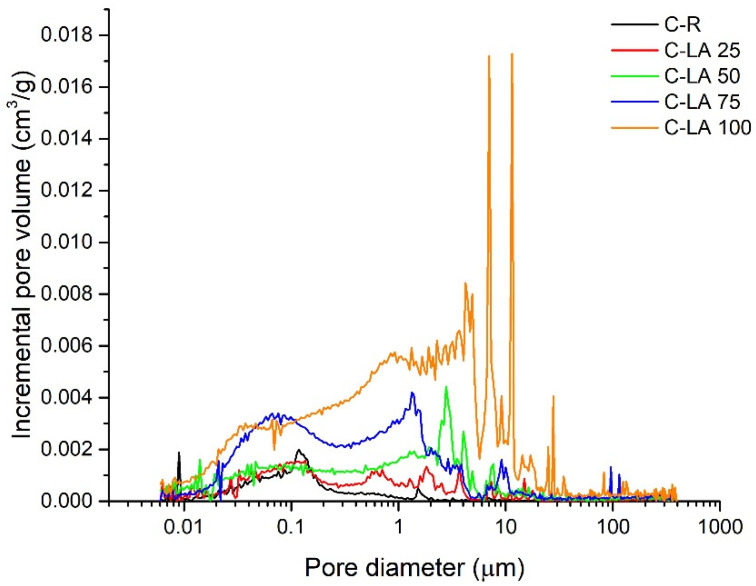
Pore size distribution–incremental pore volume curves measured for control (C-R) and LA dosed samples (C-LA 25–100).

**Figure 8:**
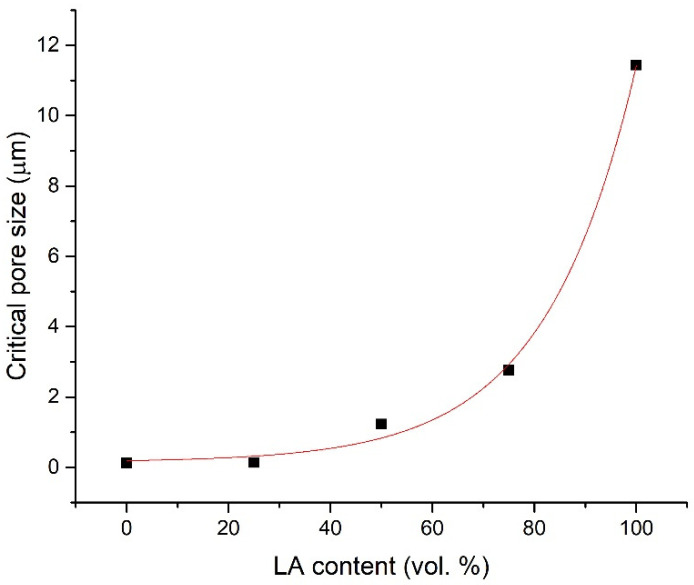
Critical pore size (d_cr_) development with regard to LBW content in concretes.

The mercury intrusion porosimetry (MIP) data measured on control samples and concretes enriched with waste aggregate are presented in Figure 6, Figure 7 and Figure 8. The graph expressing the dependence of cumulative pore volume on pore diameter (Figure 6) is divided into several intervals in shades of gray sorting pores according to their size, from gel pores up to entrained air, as was reported in [57]. The results suggest a considerable growth of pore volume for all prepared LA concretes under 10 µm in diameter in the area of capillary and gel pores. Looking closely, a more noticeable increase in pores in the given region occurs particularly under 25 vol.% of silica aggregate substitution with the alternative aggregate. Accordingly, capillary pores, in particular, in large and medium size, have a substantial impact on enhanced liquid water transport through the porous structure of building composites [58,59,60]. The evolution of critical pore size (d_cr_) in dependence on waste aggregate content in mixtures is depictured in Figure 8. The expression of critical pore size directly relates to the interconnectivity of pores (the maximum continues pore diameter) and thus on the transport rates of liquids [57,61]. According to the study conducted by Yu and Ye [62], d_cr_ is determined as the first peak of local maximum in incremental pore volume curves (displayed in Figure 7**)**. As is clearly visible in Figure 8, the values of d_cr_ show a gradually increasing exponential trend (regression coefficient of ~0.99), with values in the range of approx. 0.1 µm up to 11.4 µm. Thus, compared to the reference sample, concretes with high content of applied waste aggregate represent much more permeable material for water vapor. In addition, these composites allow more efficient transport of liquid water.

### 3.5. Liquid Water Transport

**Figure 9:**
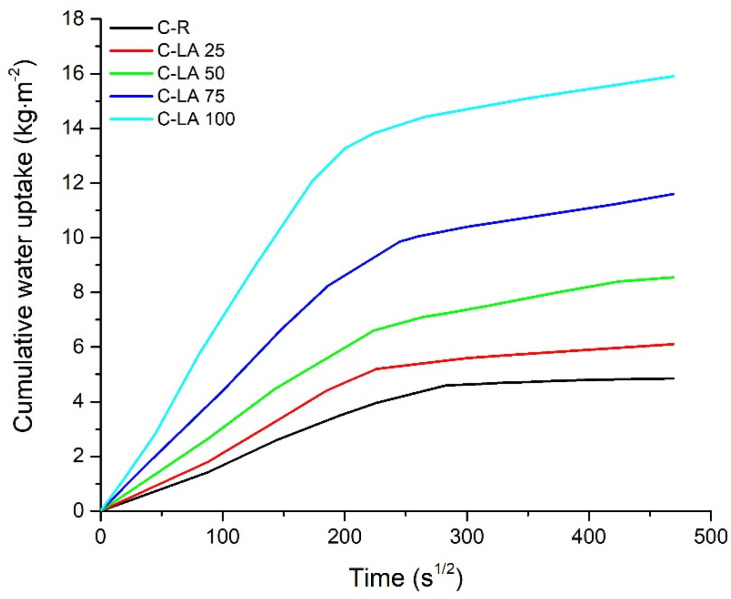
Cumulative water uptake in dependence on the square root of time for R-M and C-LA samples.

One-dimensional liquid water transport properties with dependence on LA content in hardened concretes are summarized in Table 6. The growing occurrence of lightweight waste aggregate resulted in the gradual increase of absorption coefficient (A), compared to C-R, whereas the differences were 21% (C-LA 25), 68% (C-LA 50), 121% (C-LA 75), and 211% (C-LA 100). As it is evident, hardened concrete without natural sand reached more than three times higher absorption coefficient value with respect to traditional concrete, as is clearly demonstrated in Figure 9. This fact is attributed to the considerable liquid water absorption capacity of LA versus NA (see in Table 3). The values of the absorption coefficient attributed to modern and historical bricks usually fall into the range from 0.25 to 0.39 kg·m^−2^·s^−1/2^ [46,63,64], which well correspond with data measured for lightweight concretes with the content of 25–75 vol.% of alternative aggregate.

Likewise, the increase of apparent moisture diffusivity (Χ_app_) was recorded. In comparison with control material, determined values are higher by 16% (C-LA 25), 37% (C-LA 50), 68% (C-LA 75), and 80% (C-LA 100). Compared with absorption coefficient values, percentage increments of apparent moisture diffusivities showed a growing trend as well. However, it is not so sharp. The reason for this may be found in the larger moving rate of the wet front, and thus capillary saturated water content (w_cap_) was achieved faster with increasing LA content in concretes. Such behavior could be ascribed to the higher abundance of macropores, and larger capillaries in the structure of waste aggregate modified samples [65,66]. This assumption was proofed by MIP analysis (see in Figure 6), and it was found that the number of macropores (entrained air) and larger capillaries in hardened skeleton importantly increased with the increase of silica sand alternative aggregate content in produced mixes. In addition, this hypothesis was also confirmed by critical pore size (d_cr_) analysis as described in the previous text. LA adjusted concretes with increased liquid water transport ability could be successfully applied in view of, e.g., damp suffering historical/modern masonry structures as a filling or supplementing material that allows effective moisture wicking.

### 3.6. Water Vapor Transport

Water vapor transmission properties obtained for both dry and wet cup arrangements, measured on concretes with varying silica sand and LA ratios, are presented in Table 7. Partial and total substitution of natural aggregate with the artificial LA led to the gradual increase of both water vapor permeability (δ) and water vapor diffusion coefficient (D), resulting in the reduction of water vapor resistance factor (µ). Looking closely at the obtained data, one can note differences in dry and wet cup methods: slightly lower values of water resistance factor contrasts with slightly increased values of water vapor permeability and water vapor diffusion coefficient for wet cup arrangement were recorded. Nevertheless, the decrease in water vapor resistance factor reached on average 42% (BW 25), 70% (BW 50), 79% (BW 75), and 86% (BW 100) in comparison with C-R, regardless of the dry or wet experimental arrangement. A similar observation was also reported in the work by Zemanova et al. [67], in which lightweight mineral admixture (perlite mix) was used as a natural aggregate substitution in mixes of cement–lime plasters. The presented data are in good agreement with obtained MIP results illustrated in Figure 6. The increased permeability rate may be ascribed to the high increment of capillary pores observed in C-LA concretes. According to the study presented by De Mets et al. [68], the water vapor resistance factor of brick masonry can be considered around 22. However, the water vapor resistance factor of masonry materials (brick, stones) was highly variable and may have values from approx. 9 up to those highly exceeding 50 [63,69]. All prepared C-LA concretes dispose importantly improved water vapor transmission properties and, in particular, these with waste aggregate content above 25 vol.% are in conformity with water vapor transmission behavior of masonry materials. Newly designed lightweight concretes, due to the combination of considerable water vapor permeability and the ability to effectively transport liquid water, allow the release of moisture from damp induced structures and preserves them from other water-related deterioration actions.

With respect to the above discussed experimental data, developed lightweight concretes with waste aggregate content showed high variability of determined physical properties, mechanical resistance and water/water vapor transport properties depending on the alternative aggregate content. Accordingly, the application possibilities of the proposed concretes are also varied. Whereas at a lower silica sand blend replacement by LA up 25 vol.%, composites with sufficient mechanical performance that can be used as a successful alternative to missing or highly deteriorated masonry elements/masonry parts or can serve as partial masonry reinforcement were produced. On the contrary, at lightweight aggregate dosages considerably exceeding 25 vol.%, materials with higher open porosity, lower strength, but on the other hand, disposing mitigated weight and enhanced hygric properties were prepared. Such composites may find application as lightweight filling and leveling materials, being in contact with damp-induced substrates and allowing the effective moisture transport and its gradual release by evaporation.

## 4. Conclusions

This study investigated the potential application of lightweight concrete with varying waste aggregate content as a suitable material for remediation of damp-induced and damaged masonries. At the first stage, the physical properties of raw materials intended for concretes preparation were determined and assessed. Further, structural properties, mechanical resistance, the transport of liquid water, and water vapor transmission properties were measured on hardened water cured concretes, and obtained results were compared with traditional dense concrete basically applied in remediation interventions. Moreover, the changes in the porous structure of composites caused by waste aggregate usage with the help of MIP were observed. The comprehensive investigation pointed out that the effect of alternative silica sand replacing aggregate on aforementioned properties of manufactured concretes, and several conclusions can be deduced, such as:

- Used waste aggregate compared with sand had a 10-times lower bulk density which directly led to the bulk density reduction in produced concretes, as the content of alternative aggregate increased. Concrete not containing any natural aggregate showed 2.7-times lower unit weight versus traditional concrete. As was expected, the reduced weight of hardened composites resulted in the increase of open porosity, and this increase was found to be proportional to increasing LA additions. Concrete samples with LA content of 25–50 vol.% were measured to have open porosity in the range of approx. 30–40% and such values correspond to porosities presented for different kinds of bricks and sandstones structures.
- Considering the aforementioned effect of LA additions on the open porosity of prepared concretes, mechanical properties showed a decreasing tendency. Flexural strength decreased considerably for silica sand substitutions with waste aggregate above 25 vol.%. Similarly, compressive strength development showed a substantial drop and, e.g., silica sand-free samples reached an average value slightly above 3 MPa. However, at 25 vol.% of LA additions, lightweight concretes had acceptable compressive strength performance of 29.9 MPa compared with brick and mixed masonries. Considering silica sand substitutions with LA higher than 25 vol.%, both flexural and compressive strengths noticeably decreased, and such concretes are more suitable as supplementary masonry materials replacing, e.g., mortar joints.
- One-dimensional liquid water transport ability of samples with LA content dramatically increased. The absorption coefficient average value was more than 3 times higher, and apparent moisture diffusivity was raised 1.8 times in the case of C-LA 100 samples compared to reference material. This was ascribed to considerable water absorption of waste aggregate–368%, due to the presence of cotton–polyester mix and paper-based materials in LA. In addition, partial role play also changes in the porous structure of modified concretes, as proofed MIP measurements. Nevertheless, concretes containing 25–75 vol.% of silica sand replacing material disposed of similar liquid water transport properties such as those reported for historical bricks. Samples without natural silica aggregate showed more than 7 times lower water vapor resistance factor obtained for both dry and wet cup experimental arrangements compared to ordinary concrete. Thus, all prepared concretes with a higher LA content than 25 vol.% presented a high-water vapor permeability rate, and their application as a good alternative for masonry materials (bricks, stones) can be considered.

Tested lightweight concretes are proven to be a possible alternative to traditional and historical masonry materials. Thus, composites with the waste aggregate content of 25–50 vol.% are able to ensure sufficient strength properties and comparable hydrothermal behavior like building materials used in the past. Furthermore, composites with natural aggregate substitutions above 50 vol.% with applied waste aggregate showed importantly reduced weight, and this is beneficial for their possible applications for non-bearing structures. Moreover, very effective transports of liquid water and water vapor were revealed, and thus these composites may find usage in remediation interventions of damp suffering old and/or historical buildings.

## Figures and Tables

**Table 1:** Oxide composition and material characteristics of OPC.

Oxide Composition	(wt.%)
SiO_2_	19.62
Al_2_O_3_	4.80
Fe_2_O_3_	3.34
CaO	63.70
MgO	1.42
K_2_O	0.75
Na_2_O	0.19
SO_3_	3.10
Cl^−^	0.04
Powder density (kg·m^−3^)	980
Specific density (kg·m^−3^)	3110
Specific surface area (m^−2^·kg)	408

**Table 2:** Composition of reference and LA dosed concretes.

Mix	Mass Ratio						
	CEM	:	NA	:	LA	:	WATER
C-R	1		3		-		0.5
C-LA 25	1		2.25		0.075		0.3
C-LA 50	1		1.5		0.152		0.2
C-LA 75	1		0.75		0.228		0.1
C-LA 100	1		-		0.303		-

**Table 3:** Physical properties of both aggregate types.

Substance	Loose Bulk Density (kg·m^−3^)	Compacted Bulk Density (kg·m^−3^)	Specific Density (kg·m^−3^)	Voids (%)	Water Absorption (%)
NA 0.0/0.5 mm	1 424	1 546	2 652	42	0.85
NA 0.5/1.0 mm	1 515	1 632	2 647	38	0.81
NA 1.0/2.0 mm	1 538	1 651	2 642	38	0.72
LA 0.0/0.5 mm	127	129	1 265	90	374
LA 0.5/1.0 mm	148	154	1 366	89	370
LA 1.0/2.0 mm	179	187	1 480	87	362

**Table 4:** Particle size distribution of nature aggregate (NA) and lightweight waste aggregate (LA).

Aggregate	d_10_	d_50_	d_90_
	(mm)		
NA 0.0/2.0 mm	0.20	0.72	1.67
LA 0.0/2.0 mm	0.17	1.05	1.80

**Table 5:** Mechanical resistance of reference and LA dosed concretes.

Mix	Flexural Strength (MPa)	Compressive Strength (MPa)
C-R	8.0 ± 1.4%	56.6 ± 1.8%
C-LA 25	6.5 ± 1.9%	29.9 ± 1.6%
C-LA 50	4.4 ± 1.2%	11.9 ± 0.9%
C-LA 75	3.2 ± 2.0%	7.0 ± 1.3%
C-LA 100	1.8 ± 1.7%	3.3 ± 1.4%

**Table 6:** One-dimensional liquid water transport properties.

Mix	A (kg·m^−2^·s^−1/2^)	w_cap_ (kg·m^−3^)	Χ_app_ × 10^−8^ (m^2^·s^−1^)
C-R	0.019	154.7	1.51
C-LA 25	0.023	173.8	1.75
C-LA 50	0.032	224.7	2.07
C-LA 75	0.044	277.9	2.54
C-LA 100	0.059	357.6	2.72

**Table 7:** Water vapor transmission properties.

Mix	δ × 10^−12^ (kg·m^−1^·s^−1^·Pa^−1^)	D × 10^−7^ (m^2^·s^−1^)	µ (-)	δ × 10^−12^ (kg·m^−1^·s^−1^·Pa^−1^)	D × 10^−7^ (m^2^·s^−1^)	µ (-)
	Dry Cup			Wet Cup		
C-R	1.70	2.33	113	1.93	2.57	99
C-LA 25	2.77	3.80	66	3.22	4.40	57
C-LA 50	5.34	7.30	34	6.19	8.46	30
C-LA 75	7.51	10.28	24	8.71	11.91	21
C-LA 100	11.23	15.36	16	13.01	17.80	14

## Data Availability

Comparative data used in this study are available via references links reported throughout text part.
